# Evaluating the Care Needs and Clinical and Nutritional Outcomes in Pregnant Women After Metabolic and Bariatric Surgery—A Tertiary Centre Experience

**DOI:** 10.1007/s11695-025-08315-4

**Published:** 2025-10-14

**Authors:** Brian Tran, Yi Wei Chen, Minoli Vinoda Abeysekera, Gillian Rosic, Chong Liang, Nardeen Habashy, Paik Yee Liew, Michael Devadas, Supreet Saluja, Natassia Rodrigo, Kathryn Helen Williams

**Affiliations:** 1 Charles Perkins Centre-Nepean and Sydney Medical School, The University of Sydney, Sydney, Australia; 2 Nepean Blue Mountains Family Metabolic Health Service, Department of Endocrinology, Nepean Hospital, Kingswood, Australia; 3 Departments of Upper GI Surgery, Nepean Hospital and Blacktown Hospital, Sydney, Australia

**Keywords:** Metabolic and bariatric surgery, Pregnancy, Obesity, Peri-natal care

## Abstract

**Background:**

Metabolic and bariatric surgery (MBS) is used to achieve significant and sustained weight loss. Considerable MBS are performed on women of reproductive age, with limited data regarding risks and management pre-conception and peripartum.

**Methods:**

Our retrospective audit assessed adult women in pregnancy with prior MBS, attending a specialised, multidisciplinary obesity service between January 2018 and July 2022. Baseline clinical and demographic characteristics, including surgical data, anthropometry, nutritional status, and pregnancy outcomes were collected for each pregnancy and evaluated.

**Results:**

Across 189 women and 210 pregnancies, pre-pregnancy obesity (BMI ≥ 30) prevalence was 55.9%, with polycystic ovarian syndrome and type 2 diabetes and depression and anxiety the most common associated medical and mental health problems, respectively. One-sixth of pregnancies were conceived within 12 months of MBS. Of those with available data (*n* = 174), only 43.1% (*n* = 75) had preconception dietitian reviews. Second trimester iron deficiency was noted in 64.1% (107/167) (parenteral supplementation required in 23.8% (50/210)) and vitamin B12 deficiency in 46.2% (61/132) (parenteral supplementation administered in 32.9% (69/210)). Maternal and neonatal complications occurred in 43.8% and 45.7% of pregnancies. Special care nursery (SCN) or neonatal intensive care unit (NICU) admissions were higher than background population rates.

**Conclusions:**

Women in pregnancy following MBS have complex medical and mental health backgrounds, limited pre-conception counselling and significant nutritional deficiencies, with higher rates of SCN/NICU admissions. Our study highlights the role of specialised pre-conception and perinatal services for these women.

**Supplementary Information:**

The online version contains supplementary material available at 10.1007/s11695-025-08315-4.

## Introduction

Metabolic and bariatric surgery (MBS) is a prominent intervention for achieving significant and sustained weight loss. In Australia, nearly 80% of MBS are performed on women, with a considerable number being of reproductive age [1]. MBS substantially improves fertility by promoting weight loss and restoring gonadal function and mitigates obesity-related pregnancy complications, both maternal (e.g. gestational diabetes (GDM), pre-eclampsia and other hypertensive disorders [2, 3]) and foetal (e.g. neural tube defects, hydrocephaly, cardiovascular anomalies and prematurity [4]). However, some negative outcomes have also been reported, with an increased risk of premature births and lower birth weights [2, 3].

Despite the unique challenges faced by pregnant women post-MBS, although clinical practice guidelines are available [5, 6], a comprehensive consensus is lacking, with a recent systematic review [5] demonstrating a lack of uniformity on areas such as MBS-to-conception intervals, gestational weight gain (GWG) targets, dietary composition and nutritional supplementation and surveillance required in the antenatal period. Current recommendations on perinatal management post MBS are extrapolated from general obesity and pregnancy nutritional guidelines [4, 7–10].

Literature on maternal and foetal risks associated with MBS with overweight or obesity suggest increased risk of foetal malformations (OR 1.29 [0.61–2.71]) [11], foetal loss (OR 1.31 [0.37–4.71]) [11], pregnancies complicated by premature delivery (OR 1.33 [1.01–1.75]) [11] and small-for-gestational-age (SGA) foetuses (OR 2.18 [1.41–3.38]) [11]. Studies have also demonstrated excessive gestational weight loss (39% in pregnancies < 6 months after MBS vs 2% in pregnancies > 6 months after MBS) [12], insufficient weight gain (17–73% of all women who have had MBS)[13] and impaired nutritional status with high rates of micronutrient deficiencies [13] and increased rates of caesarean Section (18.3–60.0% in those with MBS vs 14.4–28.7%) [14] when compared to women with obesity but without MBS. Several studies have indicated that suboptimal adherence to vitamin supplementation among pregnant women post-MBS is common [15].

Prospective studies, long-term follow-up and standardised reporting on pregnant women who have received MBS are scarce. Our study aims to evaluate the care needs, and nutritional and pregnancy outcomes, in a cohort who received MBS pre-conception and who attended a public multidisciplinary specialised pregnancy obesity service, based in Greater Western Sydney.

## Methods

### Study Design

This study was a retrospective audit of electronic medical records (eMR). The project was approved by the hospital district human research ethics committee (2020/ETH02669). Women aged ≥ 18 years who had undergone MBS prior to pregnancy and attended the pregnancy obesity service between January 2018 and July 2022 were identified. As data was largely descriptive, sample size was based off past similar cohort study sizes [13]. Patients without prior MBS, or who did not attend the pregnancy obesity service, were excluded. For women with multiple pregnancies, data from each pregnancy were recorded as separate entries.

### Data Collection

De-identified clinical data recorded as part of standard clinical care were retrospectively extracted from the eMR and collated in the study database, hosted on university servers using the Research Electronic Data Capture (REDCap) platform.

Baseline clinical and demographic characteristics were collected for each pregnancy, including age at time of pregnancy, Aboriginal and/or Torres Strait Islander status, country of birth, obesity-related associated medical and mental health problems, maternal anthropometrics, number of past pregnancies and live births and time from last pre-conception dietitian reviews. Active symptoms of depression were assessed using the Edinburgh Post-natal Depression Scale (EPDS), a widely recognised and validated screening tool with high accuracy [16], where a score higher than 13/30 is suggestive of depression.

MBS procedure details, including number, date and type, were noted. MBS-to-conception interval in days was calculated as described by Kjaer et al. [17] with the formula: *(date of MBS operation – date of delivery) – (gestational age in days at delivery – 14)*. This was divided by 365 then multiplied by 12 to determine the MBS-to-conception interval in months. If the exact date of the procedure was unavailable and only the year was available, then an MBS-to-conception interval range was calculated, with the date of operation being imputed as the *first calendar day (01 January)* and the *last calendar day (31 December)* of that year to calculate this range. Weight measures collected pre-pregnancy were defined as the most recent weight (either measured or self-reported) following MBS and prior to confirmation of pregnancy as documented in their first antenatal clinic (ANC) visit. GWG was calculated from the pre-pregnancy weight to the last documented weight prior to delivery. The first ANC visit was the first documented date that each participant engaged with obstetric/midwifery care.

As part of standard care [18], clinical pathology parameters (including haemoglobin, ferritin, vitamin D and active B12) were measured at various time points throughout each woman’s pregnancy. Subnormal values were considered clinically significant and requiring intervention, compared to normal or elevated values, which indicate an absence of deficiency with or without the use of supplementation. Pathology tests were first grouped according to the trimester in which they were performed; each result was then categorised as ‘low’ if they fell below the laboratory-specific reference range to account for variations across different laboratories and assays. A woman was classified as having a ‘below reference range result’ for a trimester if at least one test result was low.

Supplement use prior to, and any changes during, pregnancy was recorded. Additionally, birth and neonatal outcomes, including mode of delivery, maternal delivery complications and neonatal complications, were collected. Maternal and neonatal complications assessed are listed in Tables 5 and 6.

### Statistical Analysis

Participant demographics were reported per woman, whereas associated medical condition, clinical pathology results, neonatal complications and other pregnancy-related factors were analysed and reported for each pregnancy. Continuous variables are expressed as means with standard deviation for normally distributed values, or as median and interquartile range (IQR) for non-parametric distribution data. Categorical variables are shown as proportions. All analyses were performed using R Statistical Software (v4.4.2; R Core Team 2024). Analyses were checked by at least two authors to ensure accuracy and reduce experimenter bias.

## Results

Between January 2018 and July 2022, eMR data for 210 pregnancies in 189 women were reviewed. Most were singleton pregnancies (95.7%), with the remainder being twin pregnancies. Twenty-one (11.1%) women had two pregnancies recorded during the data collection period. The mean age was 31.8 ± 4.2 years and women were predominantly born in Australia (162/189, 85.7%), with 13 (6.9%) women identifying as Aboriginal and/or Torres Strait Islander (Table 1). Ninety-one pregnancies (43.3%) were nulliparous, with median gravidity 3 (IQR 2–4) and parity 1 (IQR 0–2). Baseline pregnancy characteristics and associated medical and mental health problems are summarised in Table 2.

**Table 1:** Demographics and metabolic and bariatric surgery characteristics of women at first antenatal clinic visit

Characteristic	Number
Total women	189
Aboriginal and/or Torres Strait Islander—*n* (%)	13 (6.7%)
Country of birth	
Australia	162 (85.7%)
New Zealand	7 (3.7%)
Other	20 (10.6%)
Number of MBS (*n* = 189)	
1	177 (93.7%)
2	12 (6.3%)
Type of MBS (*n* = 200)	
Laparoscopic adjustable gastric banding	19 (9.5%)
Sleeve gastrectomy	165 (82.5%)
Gastric bypass	16 (8.0%)
Roux-en-Y gastric bypass	13 (6.5%)
Not specified	3 (1.5%)

*MBS* metabolic and bariatric surgery

**Table 2:** Pregnancy characteristics and associated medical and mental health problems at first antenatal clinic visit

Characteristic	Number
Total pregnancies	210
Parity	
Nulliparous	91 (43.3%)
Multiparous	119 (56.7%)
Age at pregnancy—years	31.8 ± 4.2
Number of foetuses in pregnancy—*n* (%)	
1	201 (95.7%)
2	9 (4.3%)
Associated medical problems at time of pregnancy (*n* = 210)	
Polycystic ovarian syndrome	84 (44.4%)
Hypothyroidism	20 (10.6%)
Hypertension	16 (8.5%)
Gastro-oesophageal reflux disease	14 (7.4%)
Type 2 diabetes mellitus	12 (6.3%)
Hyperlipidaemia	9 (4.8%)
Metabolic dysfunction-associated steatotic liver disease	8 (4.2%)
Obstructive sleep apnoea	5 (2.6%)
Pre-diabetes	2 (1.1%)
Hyperthyroidism	2 (1.1%)
Associated mental health problems at time of pregnancy (*n* = 210)	
Anxiety	68 (36.0%)
Depression	66 (34.9%)
Eating disorders	2 (1.1%)
Documented history of nutritional deficiencies (*n* = 210)	
Iron deficiency	63 (33.3%)
Vitamin B12 deficiency	17 (7.4%)
Vitamin D deficiency	14 (7.4%)
Initial EPDS at first antenatal clinic visit with score ≥ 13 (*n* = 208)	29 (9.1%)
MBS-to-conception interval—months, median	
Last calendar day*	26.2 (IQR 14.3–50.0)
First calendar day*	26.4 (IQR 14.4–51.0)
Time from last MBS to pregnancy (first calendar day*)—months	
< 12	36 (17.1%)
12 to < 24	58 (27.6%)
24 to < 36	40 (19.0%)
36 to < 48	17 (8.1%)
48 to < 60	19 (9.0%)
≥ 60	40 (19.0%)

*EPDS* Edinburgh Post-natal Depression Scale, *MBS* metabolic and bariatric surgery.^*^Where only a year for MBS was available, the interval was calculated with the date of surgery being the first (01 January) or last (31 December) calendar day

Multi-morbidity (defined as two or more associated medical problems) was present in 98 (46.7%) pregnancies. Depression and anxiety were the most common mental health complications (Table 2), with 54 (25.7%) women having both. The median EPDS score at the first ANC visit was 5 (IQR 2–8), with 29/208 (13.9%) having a score ≥ 13.

In 179 (85.2%) pregnancies with an available height and pre-conception weight, 55.9% (100/179) had BMI-defined obesity pre-pregnancy (Table 3), with 77/179 (43.0%) having GWG above the Institute of Medicine Gestational Weight Gain Guideline recommendations [19]. Median pre-pregnancy weight and BMIs and median weight at first ANC visit are summarised in Table 3.

**Table 3:** Weight and BMI characteristics pre-pregnancy and at first antenatal clinic visit and GWG

Characteristic	Value
Weight—kg	
Pre-pregnancy (*n* = 194)	86.8 (IQR 75.5–104.0)
At first antenatal clinic visit (*n* = 185)	92.3 (IQR 79.5–106.7)
BMI—kg/m^2^	
Pre-pregnancy (*n* = 181)	32.0 (IQR 26.4–38.6)
At first antenatal clinic visit (*n* = 185)	32.1 (IQR 28.0–39.3)
Gestational weight gain (GWG)—kg (*n* = 192)	10 (IQR 5.2–15.3)
GWG by BMI (kg/m^2^) (*n* = 179)	
< 18.5 (*n* = 1)	19.3
18.5–24.9 (*n* = 28)	14.3 ± 6.3
25.0–29.9 (*n* = 50)	12.3 ± 7.2
30.0–34.9 (*n* = 37)	7.8 ± 7.7
35.0–39.9 (*n* = 24)	8.7 ± 9.0
≥ 40.0 (*n* = 39)	8.4 ± 11.5

Pre-pregnancy weight was taken as the pre-conception weight following metabolic and bariatric surgery. Body mass index (BMI) was calculated if a height was documented. The first antenatal clinic visitwas at 16.1 weeks gestation (IQR 14.5–19.2).Gestational weight gain (GWG)is calculated based on the last documented weight just prior to delivery. Not all patients had all parameters (pre-pregnancy weight, booking in weight, weight prior to delivery and height) recorded—numbers and percentages indicate number of women who had each of these measures recorded.

A total of 200 bariatric procedures occurred across 189 women, with 11 (5.8%) women having two surgeries prior to their pregnancy. No interval MBS occurred between pregnancies. Sleeve gastrectomy was the most common procedure performed, followed by laparoscopic banding and then gastric bypass (Table 1). There were 32 (15.2%) pregnancies where only the year of MBS was available. The median MBS-to-conception interval, based on the last calendar day when only the year was available, was 26.2 (IQR 14.3–50.0) months, with 38 (18.1%) pregnancies occurring with an interval of ≤ 12 months, and 95 (45.2%) with an interval of ≤ 24 months. The median MBS-to-conception interval based on the first calendar day was 26.4 (IQR 14.4–51.0) months, with 36 (17.1%) with an interval of ≤ 12 months, and 94 (44.8%) with an interval of ≤ 24 months (Table 1 and Supplementary Table 1). In pregnancies occurring > 12 months after MBS, 75/174 (43.1%) women had a dietetics review in the 12 months prior to conception. The mean gestation for the first dietitian review with the pregnancy obesity service was 21.0 ± 5.4 weeks.

**Fig. 1:**
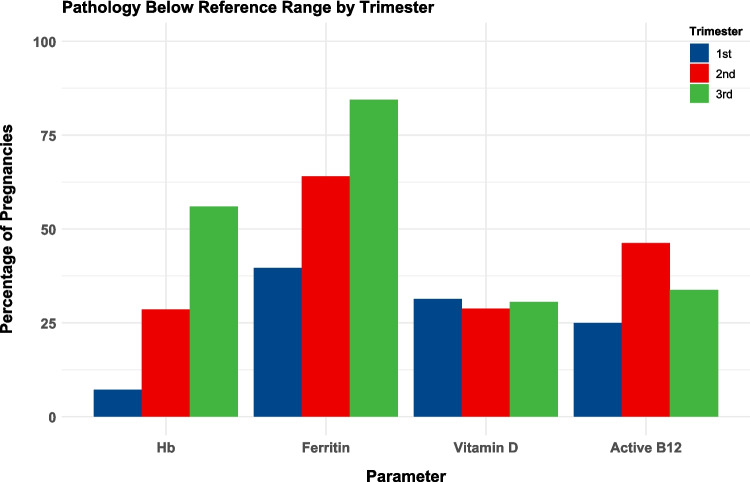
Percentage of women with pathology results below lower reference range by trimester

Tests for micronutrient deficiencies, including ferritin and vitamin D levels, as well as haemoglobin, were most frequently conducted during the second trimester (Table 4). The most common deficiency noted in the second trimester was iron, with ferritin below range in 107/167 (64.1%) pregnancies, and 61/132 (46.2%) had low active B12 levels (Fig. 1). Parenteral iron supplementation was required in 50/210 (23.8%) pregnancies, and 69/210 (32.9%) were administered parenteral vitamin B12 (Table 5).

**Table 4:** Number of pregna﻿ncies where blood testing was performed, and number of results below the reference range in each trimester

Parameter	Trimester		
Result	**1st**	**2nd**	**3rd**
Hb Below	*n* = 112/210 (53.3%) 8/112 (7.1%)	*n* = 182/210 (86.7%) 52/182 (28.6%)	*n* = 200/210 (95.2%) 112/200 (56%)
Ferritin Below	*n* = 58/210 (27.6%) 23/58 (39.7%)	*n* = 167/210 (79.5%) 107/167 (64.1%)	*n* = 167/210 (79.5%) 141/167 (84.4%)
Vitamin D Below	*n* = 51/210 (24.3%) 16/51 (31.4%)	*n* = 160/210 (76.2%) 46/160 (28.8%)	*n* = 85/210 (40.5%) 26/85 (30.6%)
Active B12 Below	*n* = 36/210 (17.1%) 9/36 (25%)	*n* = 132/210 (62.9%) 61/132 (46.2%)	*n* = 77/210 (36.7%) 26/77 (33.8%)
Corrected calcium Below	*n* = 36/210 (17.1%) 0/36 (0%)	*n* = 150/210 (71.4%) 0/150 (0%)	*n* = 71/210 (33.8%) 0/71 (0%)
Vitamin B12 Below	*n* = 43/210 (20.5%) 12/43 (27.9%)	*n* = 147/210 (70%) 57/147 (38.8%)	*n* = 49/210 (23.3%) 22/49 (44.9%)
Folate Below	*n* = 41/210 (19.5%) 1/41 (2.4%)	*n* = 157/210 (74.8%) 4/157 (2.5%)	*n* = 60/210 (28.6%) 4/60 (6.7%)
Vitamin A Below	*n* = 0/210 (0%) -	*n* = 51/210 (24.3%) 21/51 (41.2%)	*n* = 13/210 (6.2%) 9/13 (69.2%)
Vitamin E Below	*n* = 2/210 (1%) -	*n* = 49/210 (23.3%) 0/49 (0%)	*n* = 11/210 (5.2%) 0/11 (0%)
Vitamin K Below	*n* = 1/210 (0.5%) -	*n* = 50/210 (23.8%) 9/50 (18%)	*n* = 11/210 (5.2%) 1/11 (9.1%)
Zinc Below	*n* = 2/210 (1%) 1/2 (50%)	*n* = 53/210 (25.2%) 27/53 (50.9%)	*n* = 12/210 (5.7%) 8/12 (66.7%)
Selenium Below	*n* = 1/210 (0.5%) -	*n* = 49/210 (23.3%) 0/49 (0%)	*n* = 10/210 (4.8%) 0/10 (0%)
Copper Below	*n* = 1/210 (0.5%) -	*n* = 52/210 (24.8%) 1/52 (1.9%)	*n* = 12/210 (5.7%) 1/12 (8.3%)

**Table 5:** Supplement use by women at first antenatal clinic visit and the number of women commenced on supplements following this visit

Supplement	At first antenatal visit	Commenced following the first antenatal clinic visit
Multivitamin	145/210 (69.0%)	44/210 (21.0%)
Oral	145 (69.0%)	44 (21.0%)
Iron	63/210 (30.0%)	115/210 (54.8%)
Oral	63 (30.0%)	107 (51.0%)
Parenteral	-	50 (23.8%)
Vitamin D	48/210 (22.9%)	83/210 (39.5%)
Oral	48 (22.9%)	82 (39.0%)
Parenteral	-	1 (< 0.1%)
Vitamin B12	21/210 (10.0%)	94/210 (44.8%)
Oral	13 (6.2%)	36 (17.1%)
Parenteral	8 (3.8%)	69 (32.9%)
Calcium	18/210 (8.6%)	90/210 (42.9%)
Oral	18 (8.6%)	90 (42.9%)

There was a total of 219 live births at a median gestational age of 38.9 (IQR 37.9–39.7) weeks, with 25 (11.9%) occurring pre-term (< 37 weeks). There were 99 (47.1%) unassisted vaginal births and 18 (8.6%) births requiring instrumentation. The remaining 91 infants (44.3%) were delivered via Caesarean section. Maternal complications occurred in 92 (43.8%) pregnancies (Table 6). Thirty-seven (17.6%) pregnancies required perinatal management of diabetes. Neonatal complications occurred in 45.7% of pregnancies, with 45/219 (21.0%) neonates requiring an admission to the special care nursery (SCN) or neonatal intensive care unit (NICU) (Table 6). Respiratory distress syndrome (RDS) (*n* = 46, 21.9%), neonatal hypoglycaemia (*n* = 32, 15.2%) and neonatal jaundice (n = 24, 11.4%) were the most common foetal birth complications (Tables 6 and 7). While there were no stillbirths, there was one neonatal death at 12 days old in the context of extreme prematurity (gestational age 23.3 weeks).

**Table 6:** Maternal and foetal outcomes

	Study cohort	Background Australian rates
Mode of delivery (*n* = 210)		
Unassisted vaginal	99 (47.1%)	49.2%^**a**^
Instrumental	18 (8.6%)	11.6%^**a**^
Caesarean section	93 (44.3%)	39.2%^**a**^
Labour type (*n* = 210)		
Spontaneous	60 (28.6%)	41.1%^**a**^
Induced	117 (55.7%)	33.2%^**a**^
No labour	33 (15.7%)	25.6%^**a**^
Not stated	-	0.1%^**a**^
Live births (*n* = 210)		
1	201 (95.7%)	-
2	9 (4.3%)	-
Gender (*n* = 219)		
Female	110 (50.2%)	-
Male	109 (49.8%)	-
Maternal complications during pregnancy		
Diabetes in pregnancy*	37/210 (17.6%)	17.2%^**a**^
Gestational hypertension	6/210 (2.9%)	3.1%^**a**^
Maternal complications at delivery		
Post-partum haemorrhage	42 (20.0%)	-
Antepartum haemorrhage	5 (2.4%)	-
Premature rupture of membranes	5 (2.4%)	-
Post-partum hypertension	5 (2.4%)	-
Vaginal tear (3rd or 4th degree)	4 (1.9%)	2.7%^**a**^
Other	47 (22.4%)	-
Vaginal tear (1st or 2nd degree)	31 (14.8%)	54.7%^**a**^
Labial tears/grazes	10 (4.8%)	-
Episiotomy	7 (3.3%)	24.0%^**a**^
Foetal complications at delivery		
Respiratory distress syndrome	46 (21.9%)	-
Neonatal hypoglycaemia	32 (15.2%)	-
Neonatal jaundice	24 (11.4%)	-
Small for gestational age	13 (6.2%)	-
Large for gestational age	12 (5.7%)	-
Sepsis/suspected sepsis	9 (4.3%)	-
Neonatal hypothermia	3 (1.4%)	-
Meconium aspiration	2 (1.0%)	-
Birth defects	1 (0.5%)	-
Shoulder dystocia	1 (0.5%)	-
Other	10 (4.7%)	-
NICU/SCN admission	45 (21.0%)	17.0%^**ab**^
NICU admission	40 (19.0%)	-
SCN admission	5 (2.4%)	-

*NICU* neonatal intensive care unit, *SCN* special care nursery. ^*^Diabetes in pregnancy diagnosis encompassed all active diagnoses of diabetes, including gestational diabetes, type 1 diabetes, type 2 diabetes and other diabetes. ^**a**^Australia-wide rates from the Australian Institute of Health and Welfare National Perinatal Data Collection annual update 2022—data visualisation tables[32].  ^b^Rate represents combined SCN/NICU admissions, no individual rates available

**Table 7:** Neonatal intensive care unit admissions

Reason for admission (*n* = 40)	Infants (%)
Respiratory distress syndrome	26 (65.0%)
Neonatal hypoglycaemia	12 (30.0%)
Small for gestational age	5 (12.5%)
Neonatal jaundice	5 (12.5%)
Large of gestational age	1 (2.5%)
Birth defects (including neural tube defects)	1 (2.5%)
Meconium aspiration	1 (2.5%)
Other	20 (50.0%)
Prematurity	12
Sepsis/suspected sepsis	6
Hypothermia	2

## Discussion

Women who have had previous MBS and attended a specialised pregnancy obesity service in Greater Western Sydney from January 2018 to July 2022 had high rates of medical and mental health problems and low rates of pre-conception planning. Further, they had high rates of nutritional deficiencies requiring intervention and almost half had maternal and foetal complications. More than half of the pregnancies had BMI-defined obesity pre-pregnancy, using their last recorded weight before conception.

Patients with obesity have higher risks of associated cardiometabolic problems and mental health conditions [20]. In our cohort, one-fifth of pregnancies occurred in women with two or more associated medical problems. Mental health conditions were common, with over one quarter of pregnancies occurring in women diagnosed with both anxiety and depression. The proportion of patients in our cohort with depression (36%) was higher than the lifetime prevalence in the general female population (15%) [21]. Maternal perinatal anxiety and depression [22] have been associated with complications including pre-term birth, low birth weight infants and disrupted maternal-infant bonding. Pre-existing depression, anxiety and elevated BMI are all independent risk factors for the development of perinatal depression and anxiety [23]. The presence of these risk factors within our cohort highlights the need for preconception assessment and support for medical and mental health conditions in women post-MBS.

The median time from MBS-to-conception interval was longer in our cohort at 26.2 months when compared to a study by Eccles-Smith and colleagues [2], which reported a median time from MBS-to-conception interval of 20.4 months in 1282 women. In our study, almost one in two had established pregnancies within 24 months of MBS. Some studies have shown an association between surgery-to-birth intervals of < 2 years and an increased risk of prematurity, SGA and NICU admission [24], although these findings are inconsistent across literature [25]. Nonetheless, given international consensus recommendations that pregnancy should be delayed until women achieve a stable weight (typically 1–2 years post-MBS) [10], there is a need to provide contraception planning to women at risk of pregnancy when they are having MBS.

Micronutrient deficiencies have been reported widely in pregnancy after MBS, with iron and vitamin B12 deficiencies particularly common. Severe nutrient deficiencies in pregnancy may be associated with serious complications including congenital defects such as neural tube defects with low vitamin B12 and anaemia, low birth weights, preeclampsia, preterm birth and delayed growth and development with either B12 or iron deficiency [26]. This highlights the importance of screening throughout pregnancy in women who have had MBS.

Not all women in our cohort had nutritional blood tests performed in each trimester, which raises the potential for under-reporting of nutritional deficiencies. This likely reflects the lack of guideline implementation in real-world hospital settings [18, 27]. Current recommendations propose close monitoring and optimisation of diet and nutrition pre-conception and throughout pregnancy, with repeat pathology testing at least once a trimester, allowing for early supplementation when necessary [18]. These guidelines were only published in 2021 and thus were unlikely to guide the medical management in our cohort, who were recruited between January 2018 and July 2022. Other likely reasons for inconsistencies include differences in clinical decision making among providers, costs to patient [27, 28] (given restrictions on micronutrient measures in Australia, with many women having pathology outside of the hospital system during COVID) and assay limitations [27, 29] (including issues with specimen processing and reference ranges for some measures, such as vitamin A [29]).

Studies have noted that post-MBS supplementation compliance is suboptimal at 30–45%, with 20–32% of patients reporting stopping or never taking their recommended supplements at 1 year after surgery [30]. There was limited pre-conception nutritional optimisation among our cohort, with less than half receiving dietitian reviews in the 12 months preceding pregnancy or taking recommended supplements at conception.

Notably, our study has demonstrated improvements in multivitamin supplementation use, with 90% (189/210) of pregnancies maintaining or commencing on multivitamins following engagement with the pregnancy obesity service, an increase from 69% (145/210) at the first ANC visit. While rates of supplementation in our cohort significantly improved after initial contact with the pregnancy obesity service, one in ten women were still not meeting recommendations for supplementation during her pregnancy. This highlights the value of a specialised bariatric perinatal service to improve supplement adherence and reduce nutritional deficiencies in women seeking pregnancy after MBS and to ensure ongoing adequate use. Iron deficiency anaemia is a particularly common finding in women who become pregnant after MBS [26]. Comparable to our data, studies have found ferritin and haemoglobin significantly decrease in pregnant women after MBS, with persistent iron deficiency in the third trimester despite high rates of oral supplementation [26]. There were high rates of parenteral iron supplementation use in our cohort, often administered after an inadequate result from oral supplementation. As such, parenteral supplementation may be better given at an earlier time in pregnancy when there are established deficiencies than is currently recommended in clinical guidelines.

Maternal and neonatal complications were present in almost half of the pregnancies in our cohort. Our rates of admission to SCN or NICU (21%) were elevated compared with the background Australian rates of 17% [31], comparable to admission rates for infants born to mothers with obesity (20%) [32], but lower compared to mothers with diabetes in pregnancy (27.3%) [33]. Rates for RDS and neonatal hypoglycaemia in our cohort were also elevated at 21.9% and 15.2%, respectively, with the previous studies suggesting the prevalence of RDS is typically around 7%, and neonatal hypoglycaemia 3–29% in the background population [34]. Our findings suggest that pregnancies after MBS are of higher risk than the background population and would benefit from increased monitoring in the antenatal period, ideally in specialised services.

Our study had several limitations, potentially affecting the interpretation of findings. Due to the retrospective nature of the study, there were partially complete data across trimesters. Pre-MBS characteristics (including BMI, weight and obesity-related complications) were not available for collection and analysis. This was also a single-cohort study with no control group. Given that pregnant women with obesity are a high-risk group in and of themselves, further studies are required to better determine if the nutritional outcomes and complications associated with pregnancy and obesity differ in pregnant women with obesity who have and who have not undergone MBS. Furthermore, we recognise that our approach to calculating the MBS-to-conception interval for pregnancies where only the year of MBS was available may overestimate or underestimate the true interval by as much as ± 6 months. However, when assessing this interval by group, there was no statistically significant difference in the groups between the two methods (*p*-value = 0.997) (Supplementary Table 1). The different assays used to report micronutrient parameters resulted in limited inferential statistical analysis. This is compounded by incomplete datasets and standardised pregnancy reference ranges for many micronutrients (Table 4), further limiting analysis. Despite these limitations, we were able to analyse a large, high-risk group attending a specialised pregnancy obesity service, highlighting the needs of this vulnerable cohort. Our study was conducted at a single tertiary centre without a control group, potentially limiting generalisability. However, as obesity rates in our region align with those in Australia [35, 36] and other Western countries, our findings may still be relevant to similar populations. Prospective multi-centre studies would further inform clinical guidelines in this setting and evaluate the impact of specialised obesity services on improving outcomes.

## Conclusion

In our study, women who conceive following MBS often have complex medical and mental health backgrounds, limited pre-conception counselling and high rates of nutritional deficiencies compared to the background population. Our results highlight the potential role of specialised pre-conception and perinatal services for these women. Future prospective studies comparing pregnant women with obesity who have not yet undergone MBS to those who have had MBS would build on our results and guide the further development of evidence-based clinical guidelines for pregnancy after MBS, including micronutrient supplementation protocols, and support the implementation of these guidelines in real-world settings.

## Supplementary Information

Below is the link to the electronic supplementary material.

ESM1 (PDF 16.6 KB)

## Data Availability

The data that support the findings of this study are available from the corresponding author, K.H.W., upon reasonable request.
