# Supplementary material for: Evaluating the Care Needs and Clinical and Nutritional Outcomes in Pregnant Women After Metabolic and Bariatric Surgery—A Tertiary Centre Experience
Source: Obes Surg. 2025 Oct 14;35(12):5013–22. doi: 10.1007/s11695-025-08315-4 (PMC12722479; doi:10.1007/s11695-025-08315-4)
Supplement: Supplementary file 1 — (PDF 16.6 KB) [file 11695_2025_8315_MOESM1_ESM.pdf]

Supplementary Tables and Figures

Supplementary Table 1 | MBS-to-conception intervals*.

	First calendar day	Last calendar day
**Time from last MBS to pregnancy – months**		
**≤12**	36 (17.1%)	38 (18.1%)
**12 to ≤24**	58 (27.6%)	57 (27.1%)
**24 to ≤36**	40 (19.0%)	41 (19.5%)
**36 to ≤48**	17 (8.1%)	18 (8.6%)
**48 to ≤60**	19 (9.0%)	20 (9.5%)
**>60**	40 (19.0%)	36 (17.1%)
	p = 0.997	

Notes *| *Where only a year for MBS was available, the interval was calculated from the first (01 January) or last (31 December) calendar day of that year.*

Abbreviations: *MBS (metabolic and bariatric surgery)*
